# Comment on “Allogeneic umbilical cord-derived mesenchymal stem cell transplantation for treating chronic obstructive pulmonary disease: a pilot clinical study”

**DOI:** 10.1186/s13287-020-01859-5

**Published:** 2020-08-05

**Authors:** Talita Stessuk, João Tadeu Ribeiro-Paes

**Affiliations:** São Paulo State University, UNESP – Campus de Assis, Assis, Brazil

**Keywords:** COPD, Cell therapy, Clinical trial, Pulmonary emphysema

## Abstract

In the clinical study by Le Thi Bich et al., allogeneic expanded umbilical cord-derived mesenchymal stem cells (UC-MSCs) were intravenously infused to treat patients with chronic obstructive pulmonary disease (COPD). No severe or significant adverse effects were observed, while a significant improvement in COPD patients’ quality of life was reported up to 6 months. In addition, the authors argue that bone marrow-derived cells are not suitable to treat COPD based on the “failure” of 3 clinical trials (NCT01110252, NCT01306513, and NCT00683722). In fact, Le Thi Bich et al. and the three above-mentioned studies reported similar clinical outcomes, id est., no significant improvement in the pulmonary function of COPD patients. Therefore, since no COPD treatment involving cells either from bone marrow or umbilical cord was detrimental or provided lung regeneration in human patients, in our view, it is too early to point failures of cellular sources. Instead, it is a valuable opportunity to reflect on the poorly understood therapeutic mechanism of MSCs and the pathophysiology of COPD. In respect of cellular sources, only controlled trials with a strict comparison between different tissues might determine the suitability and efficacy of specific cell types to treat COPD. Finally, further studies are still required to determine whether and via which mechanism MSCs are able to provide structural and functional restoration of gas exchange in COPD patients.

## Main

In the study reported by Le Thi Bich et al., allogeneic umbilical cord-derived mesenchymal stem cells (UC-MSCs) were intravenously infused into patients with chronic obstructive pulmonary disease (COPD) [1]. The authors aimed at evaluating the safety and efficacy of using expanded UC-MSCs to treat COPD. The patients were followed up for 6 months and no severe or significant adverse effects were observed. Clinical outcomes such as number of COPD exacerbations, modified Medical Research Council (mMRC) score, and COPD assessment test (CAT) were significantly decreased overtime. However, pulmonary function parameters, exercise performance, and C-reactive protein (CRP) remained unchanged. The authors attributed the improvement in specific clinical outcomes to a “downregulated inflammation” promoted by UC-MSCs, while CRP levels could not reach statistical significance “possibly because of the small number of patients” (*n* = 20). Moreover, while the authors outlined several advantages of UC-MSCs over other tissue sources, it is argued in the introduction and discussion sections that bone marrow-derived cells are not suitable to treat COPD. Le Thi Bich et al. point out that the “failure” of 3 clinical trials (NCT01110252, NCT01306513, and NCT00683722), which used bone marrow-derived cells for COPD treatment [2–5], “revealed some issues relating to MSC transplantation for COPD.” Hence, we have few comments.

First and foremost, we share great enthusiasm regarding the reported safety on the systemic administration of allogeneic expanded UC-MSCs into a comorbid population of moderate-to-severe COPD patients. We also applaud the authors’ efforts at conducting a pilot study whose therapeutic approach lead to an improvement in COPD patients’ quality of life in 6 months. Nonetheless, our divergencies arise at discarding bone marrow-derived cells for COPD treatment due to the “failure” of previous studies.

Despite being added by Le Thi Bich et al. alongside studies which worked on isolated and expanded bone marrow-derived mesenchymal stem cells (BM-MSCs), in NCT01110252, we used neither cultured nor isolated mesenchymal stem cells (MSCs). We intravenously infused autologous fresh-isolated bone marrow mononuclear cells (BMMCs) into 4 COPD patients and reported the main results in the first worldwide publication using BMMCs as a new therapeutic approach in COPD [2]. In fact, the overall clinical outcome observed in NCT01110252 was similar to Le Thi Bich et al. and the other two above-mentioned studies, id est., no significant improvement in the pulmonary function of COPD patients [2–5]. Notably, NCT01110252 and NCT01306513 are phase 1 clinical trials which aimed at evaluating the safety and feasibility of cellular infusions in patients with severe COPD. Both studies demonstrated that autologous bone marrow-derived cells treatment in severe pulmonary emphysema is safe and free of adverse effects [2, 3, 5]. In NCT01110252, the patients were followed up over 3 years and there was an overall slowdown in the process of pathological degeneration, which means a change in the natural history of pulmonary emphysema [3].

Over the past 70 years, extensive research on bone marrow transplantation initiated and underpinned the broader use of bone marrow-derived cells in regenerative medicine. Despite being associated with an invasive procedure, autologous and allogeneic bone marrow-derived cells have been largely investigated in lung diseases either in animal models or human patients. It is only recently, however, that stem and progenitor cells from other less invasive sources such as adipose tissue, umbilical cord, and cord blood have been isolated [6]. More specifically in the 2000s, UC-MSCs were being isolated and characterized when the bone marrow-based clinical trials for COPD treatment (NCT01110252 and NCT00683722) were designed in the USA [4] and Brazil [2]. In the Netherlands, even the most recent BM-MSCs-based study (NCT01306513) was designed and followed strict ethical and regulatory legislation only available for bone marrow-derived cells [5].

Since no COPD treatment involving cells either from bone marrow or umbilical cord was harmful or provided evidence of lung regeneration in human patients, in our view, it is too early to point failures of specific cellular sources. Instead, it is a valuable opportunity to reflect on the poorly understood therapeutic mechanism of MSCs and the pathophysiology of COPD. Encouraging findings show that UC-MSCs exhibit stronger immunomodulatory capacity than BM-MSCs in vitro [1]. However, additional studies are necessary to elucidate if differences in MSC immunomodulation are reproduced in vivo. Moreover, whether MSCs act as mere anti-inflammatory agents or also provide benefits for lung morpho-functional regeneration remains to be determined.

COPD leads to a permanent pulmonary and systemic inflammatory condition besides lung degeneration. Upon systemic infusion, MSCs are primarily hosted in the lungs due to the capillary system and associated inflammatory microenvironment. As mentioned by Le Thi Bich et al., MSCs may exert anti-inflammatory control by releasing cytokines and paracrine factors which act on lung resident immune and inflammatory cells. However, MSCs undergo clearance from the lungs after few days [7]. It challenges the question whether multiple MSCs transplantations would augment lung regeneration.

In an experimental elastase-induced emphysema model, two doses of MSCs enhanced anti-inflammatory control and lung repair compared to one single dose of MSCs [8]. In COPD patients, four infusions of BM-MSCs provided a reduction in circulating CRP and when combined with lung volume reduction surgery, two infusions increased the expression of CD31, an indication of responsiveness in microvascular endothelial cells [4, 5]. Despite lacking single-infusion control groups, these clinical studies suggest that multiple BM-MSC infusions may provide anti-inflammatory effect. All cell-based clinical studies aimed at moderate-to-severe COPD patients and included similar standards for patient selection. Nevertheless, in spite of different cellular sources, the clinical trials differ considerably on treatment regime which can contribute to potential biases for the purpose of comparison. In NCT01110252, we included granulocyte colony-stimulating factor (G-CSF) to stimulate the proliferation of hematopoietic stem cells in the bone marrow. Previous results had indicated that G-CSF treatment prior to bone marrow harvest could increase the collection of CD34^+^ cells, which might be involved in tissue regeneration. However, G-CSF treatment did not provide an increase in CD34^+^ and CD133^+^ cells and we suggested the omission of G-CSF from future clinical studies [2]. Therefore, further investigation is still required to clear up the disparities in outcome between pre-clinical studies and clinical trials, as well as the exact extension of MSCs contribution to lung regeneration and if multiple cell transplantations are required in COPD patients.

Another major concern refers to the quality and existence of endogenous progenitors which could be stimulated by MSCs at the lungs of COPD patients. Although the interruption of tobacco smoking stimulates mitotically quiescent cells to replenish the bronchial epithelium [9], it is not clear the longevity of such cells in COPD patients. In addition, COPD has been related to the apoptosis of alveolar type 2 progenitor cells [10]. Most of the moderate-to-severe COPD patients enrolled in cell therapy clinical trials quit smoking over several years; hence, it must be investigated the viability of endogenous progenitor cells in COPD patients and if MSCs still would have some positive effect on such cells.

## Conclusions

In our view, the utmost caution should be exercised when designing further cell therapy clinical trials to treat COPD. In respect of cellular sources, only controlled trials with strict comparison between different tissues can determine the suitability, failure, or efficacy of specific cell types to treat COPD. Moreover, the combination of cells from different sources might be studied in order to elucidate the possibility of synergistic or additive effects. The results provided so far by cell therapy clinical trials are positive regarding safety aspects and the absence of adverse effects. Even though promising results from cell therapy-based clinical trials and animal models are encountered, several biological aspects must be investigated to elucidate the lack of functional and structural restoration of gas exchange in COPD patients. For this reason, a critical evaluation on the pathophysiology of COPD and cell therapy regimen must be considered in further randomized and controlled clinical trials.

## Data Availability

Not applicable.

## Abbreviations

BMMCs: Bone marrow mononuclear cells
BM-MSCs: Bone marrow-derived mesenchymal stem cells
CAT: COPD assessment test
COPD: Chronic obstructive pulmonary disease
CRP: C-reactive protein
mMRC: Modified Medical Research Council
MSCs: Mesenchymal stem cells
UC-MSCs: Umbilical cord-derived mesenchymal stem cells
